# Supplementary material for: The Systems Biology Research Tool: evolvable open-source software
Source: BMC Syst Biol. 2008 Jun 29;2:55. doi: 10.1186/1752-0509-2-55 (PMC2446383; doi:10.1186/1752-0509-2-55)
Supplement: Additional file 1 — SBRT Archive. An archive of the current version of the Systems Biology Research Tool. [file 1752-0509-2-55-S1.zip › sbrt-1.4.0/doc/developers_guide/api/sbrt/kernel/fba/package-use.html]

Uses of Package sbrt.kernel.fba


|  |  |  |
| --- | --- | --- |
| **Overview** \| **Package** \| Class \| **Use** \| **Tree** \| **Deprecated** \| **Index** \| **Help** | |  |
| PREV   NEXT | **FRAMES**    **NO FRAMES**     **All Classes** |  |


---


## **Uses of Package sbrt.kernel.fba**

| Packages that use sbrt.kernel.fba | |
| --- | --- |
| **sbrt.kernel.fba** | Provides classes and interfaces for creating objects relevant to Flux Balance Analysis. |
| **sbrt.kernel.fba.comp** | Provides classes and interfaces for performing Flux Balance Analysis computations. |
| **sbrt.kernel.fba.proc** | Provides classes and interfaces for executing Flux Balance Analysis processes. |
| **sbrt.shell.io.fba** | Provides classes and interfaces for reading and writing files relevant to Flux Balance Analysis. |
| **sbrt.shell.mng.fba** | Provides classes and interfaces for creating Flux Balance Analysis process managers. |
| **sbrt.shell.proc.fba** | Provides classes for converting FBA-related files of different formats. |
| **sbrt.shell.text.chem** | Provides classes and interfaces for defining formats for chemistry related objects. |
| **sbrt.shell.text.fba** | Provides classes and interfaces for defining formats for objects relevant to Flux Balance Analysis. |

Classes in sbrt.kernel.fba used by sbrt.kernel.fba

****AbstractRxn****
  
          This class is a skeleton implementation of ChemicalRxn.

****CatalyzedFluxome****
  
          This interface is used to represent fluxomes with catalyzed reactions.

****ChemRxn****
  
          This interface is used to represent simple chemical reactions.

****ExchangeRxn****
  
          This class is used to represent exchange reactions in Flux Balance Analysis.

****FluxConstraints****
  
          
This class is used to group a set of flux constraints used in
Flux Balance Analysis.

****Fluxome****
  
          This interface is used to represent fluxomes for use in Flux Balance Analysis.

****IrrevRxn****
  
          
This class is used to represent irreversible chemical reactions.

****IrrevRxnFluxome****
  
          This class is used to represent fluxomes that contain only irreversible
reactions.

****IrrevRxnFluxomeV1****
  
          This class is a concrete implementation of IrrevRxnFluxome.

****SinkRxn****
  
          This class is used to represent sink reactions in Flux Balance Analysis.

****SourceRxn****
  
          This class is used to represent chemical species sources in Flux Balance
Analysis.

Classes in sbrt.kernel.fba used by sbrt.kernel.fba.comp

****Fluxome****
  
          This interface is used to represent fluxomes for use in Flux Balance Analysis.

****IrrevRxnFluxome****
  
          This class is used to represent fluxomes that contain only irreversible
reactions.

Classes in sbrt.kernel.fba used by sbrt.kernel.fba.proc

****CatalyzedFluxome****
  
          This interface is used to represent fluxomes with catalyzed reactions.

****FluxConstraints****
  
          
This class is used to group a set of flux constraints used in
Flux Balance Analysis.

****Fluxome****
  
          This interface is used to represent fluxomes for use in Flux Balance Analysis.

****IrrevRxnFluxome****
  
          This class is used to represent fluxomes that contain only irreversible
reactions.

Classes in sbrt.kernel.fba used by sbrt.shell.io.fba

****FluxConstraints****
  
          
This class is used to group a set of flux constraints used in
Flux Balance Analysis.

****Fluxome****
  
          This interface is used to represent fluxomes for use in Flux Balance Analysis.

Classes in sbrt.kernel.fba used by sbrt.shell.mng.fba

****CatalyzedFluxome****
  
          This interface is used to represent fluxomes with catalyzed reactions.

****ChemRxn****
  
          This interface is used to represent simple chemical reactions.

****FluxConstraints****
  
          
This class is used to group a set of flux constraints used in
Flux Balance Analysis.

****Fluxome****
  
          This interface is used to represent fluxomes for use in Flux Balance Analysis.

****IrrevRxnFluxome****
  
          This class is used to represent fluxomes that contain only irreversible
reactions.

Classes in sbrt.kernel.fba used by sbrt.shell.proc.fba

****IrrevRxnFluxome****
  
          This class is used to represent fluxomes that contain only irreversible
reactions.

Classes in sbrt.kernel.fba used by sbrt.shell.text.chem

****IrrevRxn****
  
          
This class is used to represent irreversible chemical reactions.

****ReversibleRxn****
  
          This class is used to represent reversible chemical reactions.

Classes in sbrt.kernel.fba used by sbrt.shell.text.fba

****CatalyzedFluxome****
  
          This interface is used to represent fluxomes with catalyzed reactions.

****ChemRxn****
  
          This interface is used to represent simple chemical reactions.

****Fluxome****
  
          This interface is used to represent fluxomes for use in Flux Balance Analysis.

****SinkRxn****
  
          This class is used to represent sink reactions in Flux Balance Analysis.

****SourceRxn****
  
          This class is used to represent chemical species sources in Flux Balance
Analysis.

---


|  |  |  |
| --- | --- | --- |
| **Overview** \| **Package** \| Class \| **Use** \| **Tree** \| **Deprecated** \| **Index** \| **Help** | |  |
| PREV   NEXT | **FRAMES**    **NO FRAMES**     **All Classes** |  |


---
